# Comparative Phylogenetic Analysis of Ancient Korean Tea ‘Hadong Cheon-Nyeon Cha (*Camellia sinensis* var. *sinensis*)’ Using Complete Chloroplast Genome Sequences

**DOI:** 10.3390/cimb46020069

**Published:** 2024-01-24

**Authors:** Doobo Shim, Seung Ho Jeon, Jong Cheol Kim, Dong-Kyung Yoon

**Affiliations:** 1 Institute of Hadong Green Tea, Hadong 52304, Republic of Korea; doobo@hgreent.or.kr (D.S.); jckim@hgreent.or.kr (J.C.K.); 2 Department of Agricultural Life Science, College of Life Science and Natural Resources, Sunchon National University, Suncheon 57922, Republic of Korea; shjeon@scnu.ac.kr; 3 Department of Southern Area Crop Science, National Institute of Crop Science, Rural Development Administration, Miryang 50424, Republic of Korea

**Keywords:** *Camellia* (L.) *sinensis*, chloroplast (cp) genome, genome assembly, genetic variations, phylogenetic analysis

## Abstract

Wild teas are valuable genetic resources for studying evolution and breeding. Here, we report the complete chloroplast genome of the ancient Korean tea ‘Hadong Cheon-nyeon Cha’ (*C. sinensis* var. *sinensis*), which is known as the oldest tea tree in Korea. This study determined seven *Camellia sinensis* var. *sinenesis*, including Hadong Cheon-nyeon Cha (HCNC) chloroplast genome sequences, using Illumina sequencing technology via de novo assembly. The chloroplast genome sizes ranged from 157,019 to 157,114 bp and were organized into quadripartite regions with the typical chloroplast genomes. Further, differences in SNPs and InDels were detected across the seven chloroplast genomes through variance analysis. Principal component and phylogenetic analysis suggested that regional constraints, rather than functional constraints, strongly affected the sequence evolution of the cp genomes in this study. These genomic resources provide evolutionary insight into Korean tea plant cultivars and lay the foundation for a better understanding of the ancient Korean tea plant HCNC.

## 1. Introduction

Tea (extract from the leaves of tea plant *Camellia sinensis* (L.) O. Kuntze) is one of the most popular beverages worldwide as it features attractive flavors and provides substantial economic value. The demand for tea production has recently increased due to its characteristic secondary metabolites, such as catechins, polyphenols, and caffeine, which have numerous human health benefits [1]. Based on morphological and physiological characteristics, the three main natural hybrids that make up the cultivated taxa of tea are *C. sinensis* (L.) O. Kuntze (also named China type), *C. assamica* (Masters) (also named Assam type), and *C. assamica* subsp. *lasiocalyx* (Planchon ex Watt.) (also called Cambod or Southern type) [2,3]. In Asia, tea plants have been grown for thousands of years. It was said to have originated in the Assam region of India and the Yunnan Province of China [4].

The origin of Korean tea, however, is not clear. Many Korean historical accounts state that tea seeds were first brought from China to Korea in the early 9th century, although widespread tea cultivation did not begin until the 12th century [5]. There is still an argument about whether there were wild tea plants native to Korea. Two types of green tea have been produced in Korea: the native variety, which comes naturally in the surrounding area of Mountain Jiri, and the cultivated type, whose breeding lines come from China and Japan [5]. Korean tea is mainly produced in Gyeongsang Province, Jeolla Province, and Jeju Island. Among these regions, the tea cultivation area near Mt. Jiri in Hadong in south Gyeongsang province and Boseong in south Jeolla province are known to be the most prominent areas. A study comparing and analyzing the different genetic resources of the wild green tea population was carried out because, among these, there is room for discussion about the genetic background of the wild tea population flourishing in the Hadong region. Consequently, the cultivated and wild populations of green tea in China and Japan originated from distinct sources compared to the cultivated variety [6]. The wild tea tree of the Hadong region named ‘Hadong Cheonnyeon Cha’ (‘Cheonnyeon’ means thousand years, while ‘Cha’ means tea in Korean), used in the previous study, is known to be the oldest tea tree in Korea, is estimated to be over 800 years old. Due to its high historical value, the ‘Hadong Cheonnyeon Cha (HCNC)’ population is managed and protected in Korea. Moreover, the genetic resources found in the native tea plants remain crucial for the breeding of green tea.

Functional genomics is now essential to understanding tea plant biology thanks to the development of sequencing technology [7,8]. The advances in next-generation sequencing technology, especially third-generation sequencing technology that produces reads longer than 10 kb, have recently led to decoding several cp genomes [9]. Because of this, even within tiny taxonomic groups, phylogenetic analyses based on cp genome data are becoming more accepted [10]. The chloroplast DNA, sometimes called the chloroplast genome, is commonly referred to as cp DNA [11]. According to Jansen et al. [12] and Palmer [13], it has the usual quadripartite structure, which is typically composed of four parts: a large single copy (LSC), a small single copy (SSC), and two inverted repeats (IRs) [12]. Plant cells or leaves typically contain 400–1600 copies of the chloroplast genome [14]. Numerous species’ taxonomic and phylogenetic analyses have profited from the effective application of the chloroplast genome sequence [15]. Additionally, there is a significant difference in the molecular evolution rate between the coding and non-coding regions [13]. However, cp DNA does not undergo genetic recombination, is highly conserved [16], and exhibits maternal inheritance [17]. Environmental variables and the chloroplast’s developmental program impact the regulation of gene expression, which occurs at multiple stages, namely transcription, post-transcription, translation, and post-translation [18]. Most of the regulation over chloroplast gene expression occurs at the post-transcriptional stage [18]. As a result, the plant chloroplast genome offers several benefits for illuminating species relationships and yielding a wealth of crucial information regarding chloroplast genetic change [19]. Because all the cp genomes have a low mutation rate and limited recombination, they are dependable sources of information for determining phylogeny and evolutionary history based on highly conserved gene content and structure. Several cp genomes have been used extensively recently, particularly in the *Camellia* genus, for phylogenetic reconstruction. Five SSR markers were found by Liu et al. [20] to be a core marker collection that is suggested for fingerprinting tea plant cultivars or accessions [20]. Consequently, whole-genome sequencing and resequencing are now valuable techniques for determining the differences between various tea kinds, mining functional genes, and researching the origins of tea plants [21]. Lee et al. [22] completed chloroplast genome sequence of the chloroplast genome sequence of the Korean *C. sinensis* cultivar Sangmok. According to their phylogenetic analysis, the *C. sinensis* L. cultivar Sangmok is closely related to KJ806277 *Camellia pubicosta* [22]. By combining the RNA-Seq data of 217 different tea accessions with high-quality chromosome-scale reference genome for an ancient tea tree (DASZ), Zhang et al. [23] were able to clarify the lineage of tea cultivars and identify the major players in the breeding of Chinese tea [23]. Furthermore, the *C. sinensis* (CSS) populations underwent a stronger selection for flavor and disease resistance during domestication than the *C. sinensis* (CSA) populations, according to the whole-genome resequencing of 139 tea accessions worldwide [21].

In this study, we complete cp genome sequences of seven *C. sinensis* var. cultivars to investigate the origin and genetic comparison between HCNC and other *C. sinensis* cultivars. Our genetic analyses suggested that HCNC might have evolved differently from the Chinese or the Japanese *C. sinensis* cultivars.

## 2. Methods and Materials

### 2.1. Plant Materials and DNA Sequencing

Fresh leaves of seven (four Korean, one Chinese, and two Japanese) specimens of C. sinensis cultivars were collected in the experimental field of the Institute of Hadong Green Tea in Korea. The leaves were immediately preserved in liquid nitrogen before DNA extraction. The total genomic DNA was extracted with the DNeasy Plant Mini Kit (Qiagen, Valencia, CA, USA), following the manufacturer’s instructions. The final DNA concentration was measured using a NanoDrop and Qubit spectrophotometer. Genome libraries (631 bp) were constructed using the Covaris S series (Covaris, MS, USA), following the manufacturer’s instructions. After purification, the extracted DNA was used to generate paired-end sequencing libraries according to the Illumina standard protocol (Illumina, San Diego, CA, USA). Genome sequencing was carried out on the Illumina Hiseq 2500 platform, following the manufacturer’s protocol (Illumina, San Diego, CA, USA). After sequencing and data treatment, 157,511,170,132–196,874,632,856 clean reads were retrieved for the seven *C. sinensis* chloroplast genomes.

### 2.2. Genome Assembly

FastQC and Sickle trimmed all of the raw reads. Next, we performed genome assembly using Trimmomatic0.38, BWA (v0.6.1), Picard 2.9.0, GATK (v4.1.3.0), and SnpEff v4.3t. The low-quality nucleotides and adapter sequences in each read were removed using Trimmomatic. The draft genome sequence of the tea plant (*Camellia sinensis*), downloaded from the database of the NCBI (https://www.ncbi.nlm.nih.gov/sra, accessed on 15 October 2023), was used as a reference genome. Using the default parameters, paired-end reads were mapped to the tea reference genome with BWA (v0.6.1) software. The Picard package was used to filter the duplicated reads. After removing duplicated reads with Picard 2.9.0, the variants in each sample were called using a GATK HaplotypeCaller. After alignment, SNP calling was conducted per individual using SAMtools. The CoverageBed program in BEDtools (v2.17.0) was used to calculate the coverage of sequence alignments. SAMtools software (v1.10.2) was used to convert mapping results into the BAM format and to filter the unmapped and nonunique reads.

### 2.3. Functional Annotation of Genetic Variants

**Figure 1:**
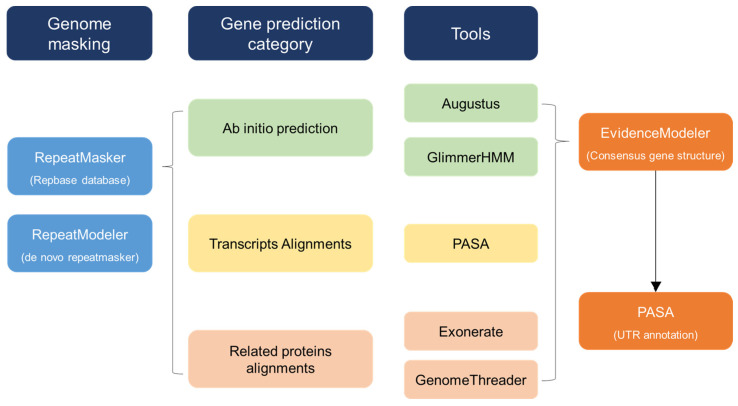
Workflow of annotation procedure. Overview of the data and workflow of the computational annotation and manual annotation.

SNP annotation was conducted based on the draft genome of *C. sinensis* using the SnpEff program. According to the annotation information, SNPs were distributed in the upstream regions, downstream regions, intergenic regions, exonic regions, 3′ UTRs, 5′ UTRs, splicing sites (which were distributed in 1 kb regions away from the transcription start site), and intergenic regions. Moreover, SNPs in exonic regions were further divided into synonymous SNPs (sSNPs) or non-synonymous SNPs (nsSNPs). The phylogenetic constructions were applied using two methods: neighbor joining and maximum likelihood based on a distance matrix calculated with MAFFT (v7.123b), MUSCLE (v3.8.31), Gblocks (0.91b), and FastTree (2.1.11). The workflow of the annotation procedure is described in Figure 1. PCA was also used to evaluate the genetic differentiation of the seven *C. sinensis* populations using the R package (RColorBrew, ggplot2, phylogram; v3.5.3). The aligned results were trimmed by trimAI v1.4. The phylogenetic analyses were implemented using maximum likelihood (ML) and neighbor-joining analysis methods based on the complete cp genome data. SNP data of the seven cp genomes sequenced in this study were used to perform PCA using GCTA v1.25.2, and the first two components were plotted.

### 2.4. Comparative Analysis and Gene Prediction

The chloroplast genome size and organization were compared, and the differences in the IR border of seven *C. sinensis* chloroplast genomes were analyzed. First, genome masking was conducted through RepeatMasker (version open 4.0.7) and RepeatModeler (version open 1.0.11). Gene prediction was performed with three categories—ab initio prediction, transcript alignments, and related protein alignments—as the tools of ab initio prediction used Augustus (Augustus 3.3) and GlimmerHMM (GlimmerHMM 3.0.4). Also, PASA (PASApipeline 2.2.0) was used for transcript alignments. Related protein alignments were performed with Exonerate (exonerate 2.2.0 x86_64) and GenomeThreader (gth 1.6.6). For consensus gene structure, they used EVidenceModeler (EvidenceModeler1.1.1). Finally, there is UTR annotation with PASA (PASApipeline2.2.0).

### 2.5. Principal Component Analysis (PCA) and Phylogenetic Analysis

Twenty-two complete chloroplast genome sequences were used in the phylogenetic analysis, including seven *C. sinensis* genome samples and fifteen completed genomes of *C. sinensis* from GenBank. All chloroplast genome sequences were aligned using the MAFFT algorithm on the MAFFT v7.123b and adjusted manually as needed gene maps of the seven *C. sinenesis* chloroplast genomes. The circular maps of seven *C. sinensis* chloroplast’s complete genomes were drawn using OrganellarGenomeDRAW v. 1.3.1 (OGDRAW) [24]. The phylogenetic reconstructions were applied using four methods: neighbor joining (NJ), maximum likelihood (ML), UPGMA, and minimum evolution (ME). Phylogenetic trees of seven *C. sinensis* were constructed using MEGA-X (Version 10.0.5) through the four methods described above.

## 3. Results

### 3.1. Chloroplast Genome Sequencing and Assembly

The chloroplast genomes of seven specimens of the *C. sinensis* cultivars were sequenced using the Illumina HiSeq 2500 system, producing clean data ranging from 157 to 196 Giga base pairs (Table 1). After achieving clean reads (98.5%), they were mapped to the complete genome, respectively. The genome of HCNC was produced using 162 Gbp clean reads, representing a total of 927 Gbp paired-end reads (Table 1). The mapped reads of seven *C. sinensis* were from 888,862,712 to 1,109,091,777 bp (Table 1). More than 24.3 billion bases from high-throughput sequencing (Q20 was 99.99%), along with the chloroplast genome of seven *C. sinensis*, were assembled according to “depth range” (≥510) and used to align with the reference GCF_004153795.1.

### 3.2. Chloroplast Genome Features of HCNC

**Figure 2:**
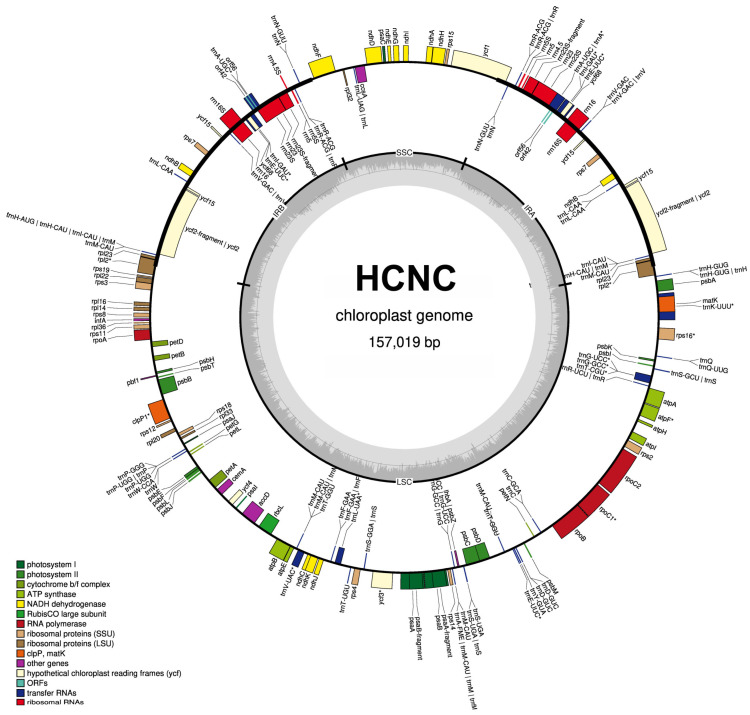
Chloroplast genome map of HCNC. Genes outside the main circle are transcribed clockwise, while genes on the inside are transcribed counterclockwise. The Organellar Genome Draw (OGDraw) online software (v 1.3.1) was used to draw this map. Different colors represent genes with other functions. The inner circle’s gray portion indicates the chloroplast genome’s GC content. *: Gene containing a single intron.

**Figure 3:**
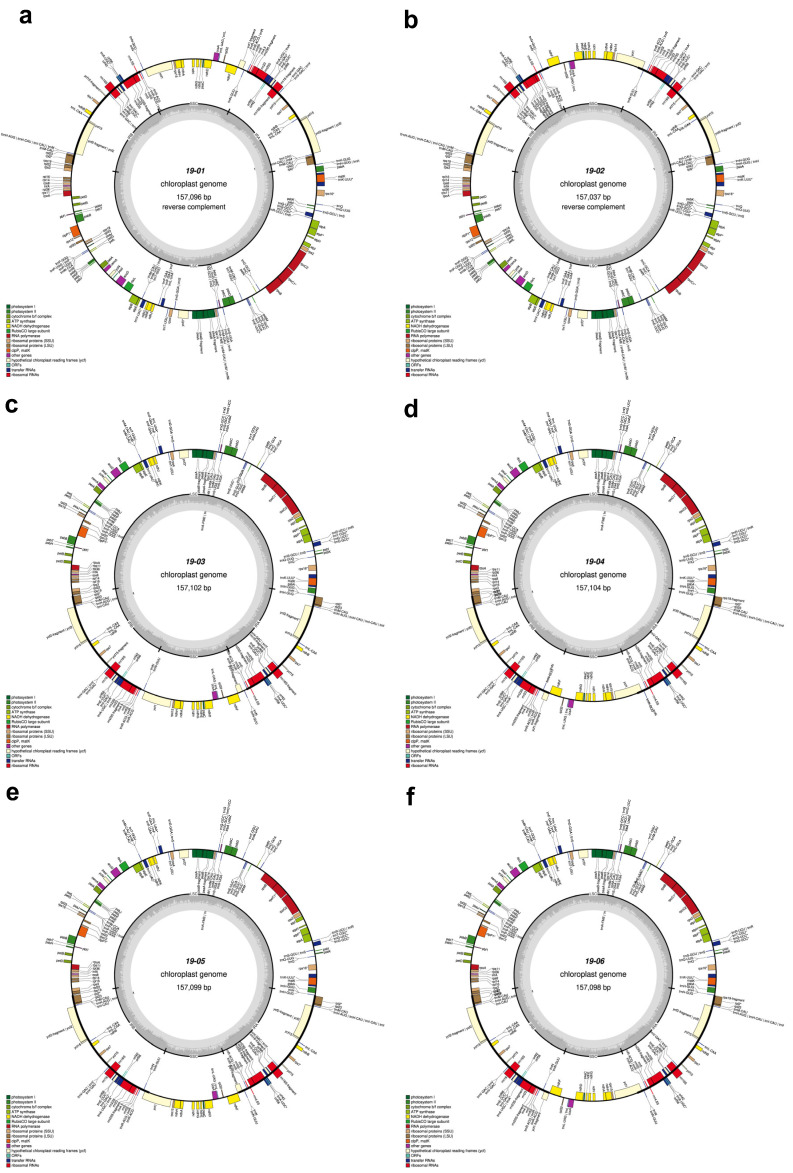
Chloroplast genome maps of six *C. sinensis* cultivars. Genes outside the main circle are transcribed clockwise, while genes on the inside are transcribed counterclockwise. The Organellar Genome Draw (OGDraw) online software was used to draw this map. Different colors represent genes with other functions. The inner circle’s gray portion indicates the chloroplast genome’s GC content. (**a**) Beachwisull; (**b**) Keumsull; (**c**) Chinese wild type; (**d**) Saemidori; (**e**) Fushun; (**f**) Hadong wild type. *: Gene containing a single intron.

The total length of seven *C. sinensis* chloroplast genomes ranged from 157,012 bp to 157,104 bp (Table 1, Figure 2 and Figure 3). All these chloroplast genomes exhibited the typical quadripartite structure, consisting of a pair of IRs separated by the LSC and SSC regions (Figure 3). The entire chloroplast genome of HCNC has four standard structural regions, which include a large single copy (LSC), a small single copy (SSC), and two reverse repeat regions (IRa and IRb) (Figure 2). The total GC content of the HCNC cp genome was 38.3%. The chloroplast genomic characteristics of HCNC were compared with six other *C. sinensis* cultivars (Figure 3). The whole genome length of HCNC was not much different from that of the other *C. sinensis* cultivars. Among them, the whole chloroplast genome size of HCNC and Chinese wild type was the closest (Figure 2 and Figure 3).

### 3.3. Gene Annotation

Each of the genomes encoded unique genes. These genes belong to several categories with different functions. They can be divided into self-replication (tRNA, rRNA, ribosome subunits, and DNA-dependent RNA polymerase), photosynthesis related (NADH oxidoreductase, photosystem subunits, rubisco, cytochrome, and ATP synthase), transcription and translation related (transcription, ribosomal proteins, translation initiation factor), biosynthesis (maturase, envelope membrane protein, acetyl-CoA subunits, translational initiation factor and protease), and conserved open reading frames. We identified 14 genes in SSCs, 29 in IRA, 22 in IRB, and 101 in LSCs in the HCNC cp genome (Table 2). Also, we detected 45 photosynthesis-related genes, 17 transcription and translation-related genes, 16 protein-coding genes, 14 tRNA genes, 4 rRNA genes, and 32 tRNA genes in the HCNC cp genome (Table 3).

### 3.4. Chloroplast Genome Sequence Variations in HCNC

#### 3.4.1. Variations in the Chloroplast Genome of HCNC

**Figure 4:**
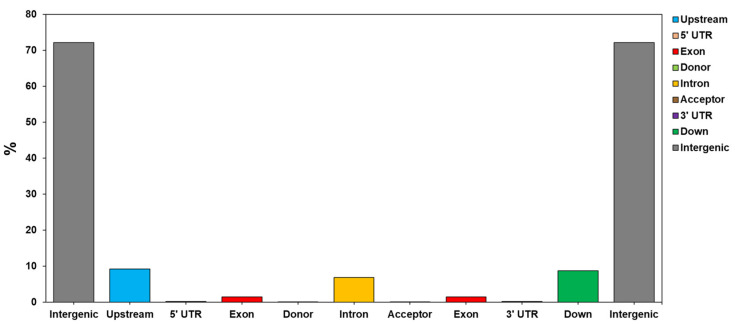
The distribution of the identified SNP in the different genomic regions (intergenic, upstream, downstream, intron, exon, and others) of the HCNC.

The seven *C. sinensis* cp genome sequences were aligned to understand the characteristics of variations through variant analysis. As a result, alternative genotypes of InDel and SNP were detected for each position (Table 4 and Table 5). InDel variations were detected to be the most numerous in the Chinese wild type, while HCNC has the most Homozygous type variations number. The SNP variation analyses revealed that the Chinese wild type was the most frequent, and HCNC has 74,542,948 SNP variations in the whole cp genome (Table 5). Table 6 and Figure 3 show a complete genome analysis, including the number and distribution of variations in each region. The region with the most distribution of variations, except for the intergenic region, was the upstream region. Variations in the exon region accounted for 1.43% of the total account (Table 6 and Figure 4).

#### 3.4.2. Comparison of Chloroplast Genome Sequence Variations

**Figure 5:**
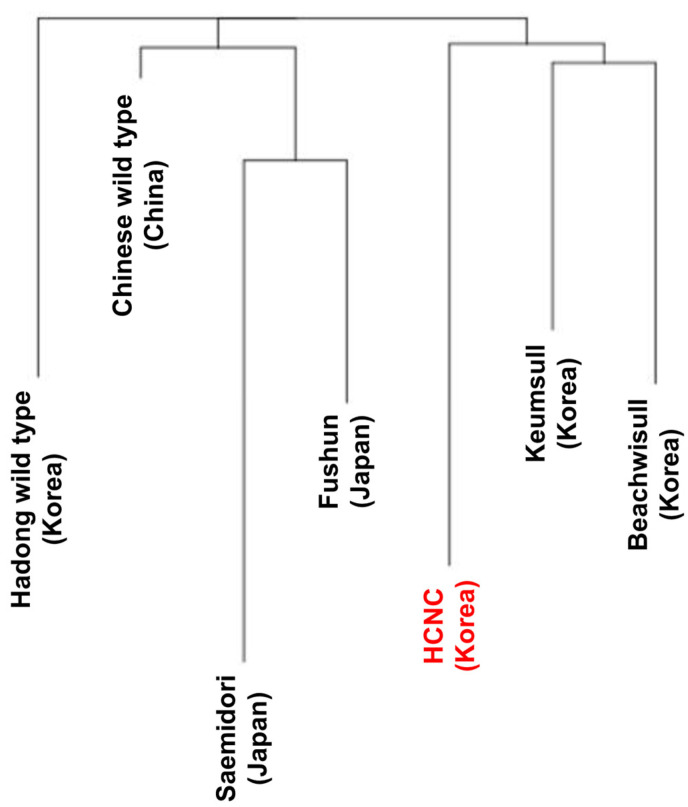
Phylogenetic trees of seven *C. sinenesis* cultivars. Phylogenetic trees were constructed using complete cp genome data with the maximum likelihood method. The red color character indicates HCNC.

**Figure 6:**
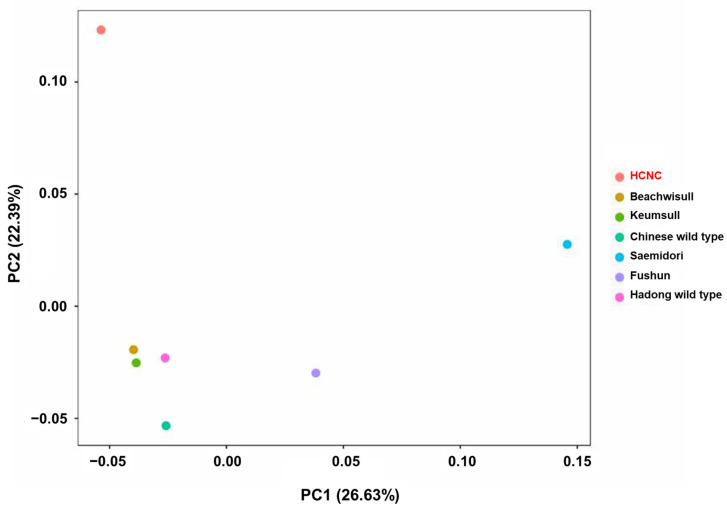
Principal component analysis (PCA) plots of the seven *C. sinensis* individuals. The legend at the right indicates the cultivars of each circle.

The SNP analysis was performed to compare and analyze genetic similarity between seven *C. sinensis* cp genome sequences. There are two types of SNP in the exon region: synonymous SNPs and non-synonymous SNPs. Synonymous SNPs usually produce the same protein even if the one base changes, whereas non-synonymous SNPs produce missense and nonsense mutations. Non-synonymous SNP analyses were performed in two ways: constructing a phylogenetic tree using obtained non-synonymous SNP data from variant annotation analysis and principal component analysis (PCA). The phylogenetic tree was constructed by neighbor-joining methods (Figure 5). According to the findings, two distinct groupings can be made up of seven cultivars: three Korean cultivars clustered into one group, while China and Japanese cultivars clustered into another. The Hadong wild type was the first to be separated from the sister clade. Using SNP data, we performed PCA to evaluate the relationships between the seven *C. sinensis* cp genomes (Figure 6). Findings suggested that there was a substantial genetic diversity among the genomes. In the PCA plot, three Korean cultivars, Beachwisull, Keumsull, and Hadong wild type, were grouped according to their geographical origin. Interestingly, HCNC is only separated by far distance from other cultivars. The Japanese cultivar, Saemidori, is divided in the opposite direction with HCNC. The Fushun, which is the Japanese cultivar, and the Chinese wild type were slightly separated from the Korean group.

### 3.5. Phylogenetic Analysis

**Figure 7:**
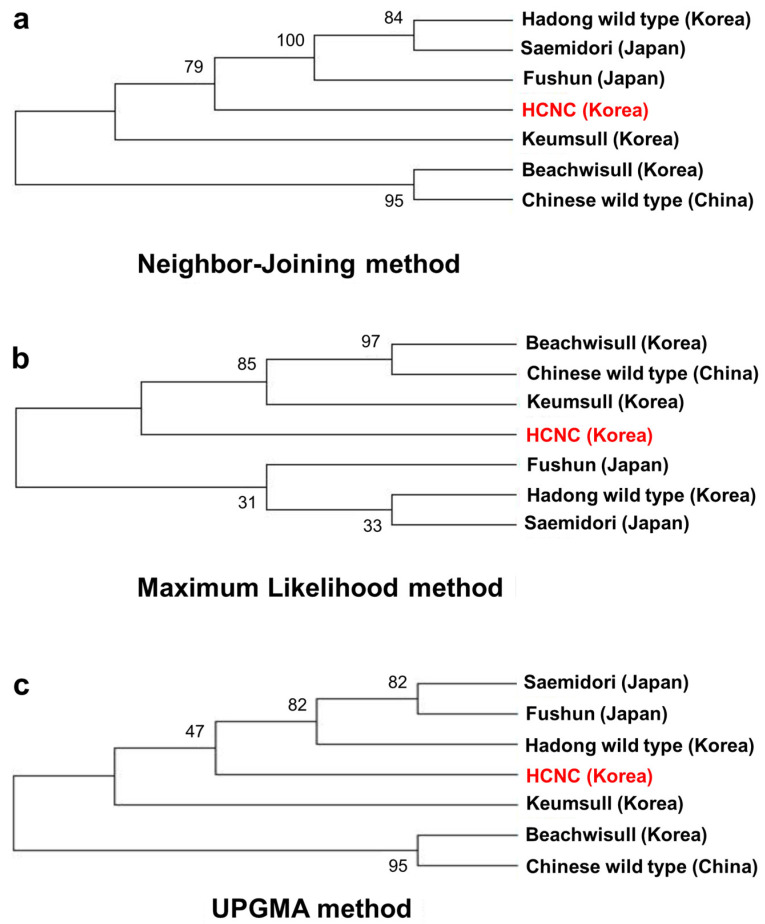
Phylogenetic trees of seven *C. sinenesis* var. *sinensis* cultivars. (**a**) Phylogenetic trees constructed using complete cp genome data with neighbor-joining method; (**b**) phylogenetic trees constructed using complete cp genome data with maximum likelihood analysis method; (**c**) phylogenetic trees constructed using maximum likelihood with UPGMA method. The red color character indicates HCNC.

Past research has shown that terrestrial plants’ chloroplast genome has been a valuable source among related species, which is applied in phylogenetic studies [25,26]. This paper aligned all chloroplast genomes of seven cultivars, including three Korean cultivars, one Chinese cultivar, three Japanese cultivars, and Korean HCNC. The phylogenetic tree was constructed using neighbor-joining, maximum likelihood, and UPGMA methods. Firstly, we identified the relationships of seven *C. sinensis* cultivars complete in this study (Figure 7a–c). The phylogenetic tree shows that HCNC was closely related to the Hadong wild type and the two Japanese cultivars in the neighbor-joining method. In maximum likelihood analysis, HCNC was more closely associated with Chinese wild type and two Korean cultivars than Japanese cultivars. The result of the UPGMA analysis was similar to the neighbor-joining method.

## 4. Discussion

**Figure 8:**
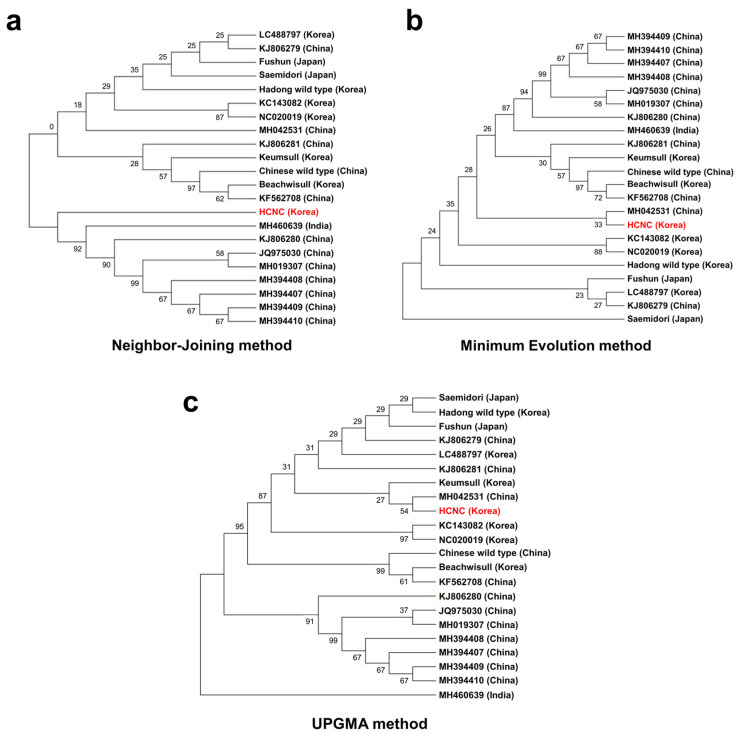
Phylogenetic trees of fifteen *C. sinenesis* varieties. (**a**) Phylogenetic trees constructed using complete cp genome data with neighbor-joining method; (**b**) phylogenetic trees constructed using complete cp genome data with maximum likelihood analysis method; (**c**) phylogenetic trees constructed using maximum likelihood with UPGMA method. The red color character indicates HCNC.

It has been proposed that the cp genome can be helpful for low taxonomic-level phylogenetic reconstructions [25,27,28,29]. Using cp genome data, the evolutionary relationships between the several species within *Camellia* (Theaceae) were clearly resolved [30,31]. In the present study, we constructed three phylogenetic trees to identify relationships between HCNC and 22 *C. sinensis*, the complete cp genomic tree, and GenBank. According to the phylogenetic relationships, the 22 *C. sinensis* were mainly clustered into two clades with the neighbor-joining method (Figure 8a–c). HCNC grouped the same clade with *C. assamica* cultivars from China and India (Figure 8a). In the minimum evolution method, HCNC tends to be closely related to the Chinese *C. sinensis* cultivar, Anhua. However, it was difficult to close relationships due to their low bootstrap value (33%). Also, *C. sinensis* cultivars are more closely related than *C. assamica* cultivars with HCNC. The Saemidori, the Japanese cultivar, has a relationship with HCNC. This result is similar to the PCA result we indicated above in Section 3.4.2. Lastly, the UPGMA method is closely related to *C. sinensis* cultivars with HCNC. Likewise, HCNC tends to be closely associated with the Chinese *C. sinensis* cultivar, Anhua. However, it was difficult to close relationships due to their low bootstrap value (54%).

Comparative analysis results indicate that seven cp genome sequences of *C. sinensis* showed highly conserved genomic structures [26,32,33]. Genes of *ycf1* and *infA* were found to be pseudogenes in HCNC. The pseudogenizations of *ycf1* and locations of *ycf1* copies are commonly found in other plants [34,35,36]. Although it was formerly believed that the pseudogene had lost its capacity to code for proteins, it is now understood to be an evolutionary remnant of the functional gene [37]. Comparative study results showed that CDS and IR areas were more conserved within cp genomes than IGS and SCs. Comparative study results showed that, within cp genomes, CDS and IRs areas were more conserved than IGS and SCs, respectively [32,38]. Because of their remarkable conservation, the IR regions help stabilize the chloroplast genome’s structure. The IR region may have expanded or contracted depending on the species, according to a comparison of the IR/SC boundary areas [39]. CP genome length changes are frequently caused by the IR regions’ expansion and contraction. According to other studies, the conservatism caused the IR regions to exhibit a lower degree of sequence divergence than LSC and SSC areas in *Camellia* cp genomes. Numerous studies have examined species identification and molecular phylogeny at the interspecific level using the polymorphic cp DNA non-coding regions [6]. Three variable areas that can be utilized for phylogenetic analysis and species identification have been identified: *petA-psbJ*, *psbI-trnS*, and *ccsA-ndhD* [6]. Chloroplast genomes have become popular in taxonomy research for assessing the relationships between closely related species [37,40]. For instance, the cp genomes of 35 species from the Ranunculaceae family, which comprise 31 genera, were sequenced and used to shed light on the long-standing systematic disputes within this family [35].

From our results and previous reports [41], we propose the following hypothesis. Considering that HCNC is close to the Chinese species, it originated in China and has since evolved and differentiated independently in Korea. In addition, it can be assumed that HCNC was differentiated first, as it shows different types from Hadong wild type and cultivated species. Our phylogenetic analyses based on seven cp genomes successfully resolved intergeneric relationships within *C. sinensis*. As demonstrated by earlier research, even with the full chloroplast genome material available for analysis, all phylogenetic relationships could not be fully resolved. Furthermore, the comparison object we selected were no plant groups involved in the other genera of the family *Theaceae*, which might provide useful information for the evolutionary study of HCNC. Future research on evolution and differentiation will be required, and investigations such as molecular biological age estimation and the examination of morphological differences in leaves are thought to be essential.

In summary, our research can promote the exchange of information between the nuclear genomes of *Camellia* species and provide valuable genomic resources for phylogenetic studies.

## 5. Conclusions

Our study provides a valuable resource for understanding ancient tea plants’ chloroplast structure, variation information, and phylogenetic relationships in Korea. The significant SNPs associated with favorable variants, selection signals, and candidate genes are a valuable resource for the further improvement of leaf traits and plant types in ancient tea plants in Korea. The complete cp genome sequence will contribute to additional molecular identification, genetic diversity, and phylogeny studies.

## Figures and Tables

**Table 1:** Sequencing data used for HCNC genome assembly.

Sample ID	Total Reads	Clean Reads (%)	Total Bases (bp)	Clean Bases (bp)	Mapped Reads (%)	Average Depth
HCNC	1,073,860,934	1,059,080,726 (98.62)	162,153,001,034	158,364,836,118 (97.66)	926,909,985 (96.15)	47.67
Beachwisull	1,177,245,210	1,157,920,230 (98.36)	177,764,026,710	172,823,184,351 (97.22)	952,928,984 (95.42)	48.91
Keumsull	1,299,116,428	1,281,585,276 (98.65)	196,166,580,628	191,706,898,728 (97.73)	1,109,091,777 (95.95)	57.07
Chinese wild type	1,043,120,332	1,028,083,462 (98.56)	157,511,170,132	153,652,236,895 (97.55)	888,862,712 (95.43)	45.66
Saemidori	1,174,595,776	1,157,020,058 (98.50)	177,363,962,176	172,986,551,338 (97.53)	979,413,130 (96.24)	50.34
Fushun	1,250,336,194	1,232,795,400 (98.60)	188,800,765,294	184,077,393,584 (97.50)	1,025,612,503 (95.75)	52.64
Hadong wild type	1,303,805,456	1,281,819,474 (98.31)	196,874,623,856	191,127,960,902 (97.08)	1,053,467,620 (95.79)	54.05

**Table 2:** List of HCNC cp genome genes organized according to their location.

Group of Genes	Name of Genes
SSC	*ccsA*, *ndhA*, *ndhD*, *ndhE*, *ndhF*, *ndhG*, *ndhH*, *ndhI*, *psaC*, *rpl32*, *rps15*, *trnL*, *trnL-UAG*, *ycf1*
IRA	*ndhB*, *orf42*, *orf56*, rpl2*, *rpl23*, *rps7*, *rrn16*, *rrn16S*, *rrn23*(×2), *rrn23S*(×2), *rrn4.5*(×2), *rrn5*(×2), *rrn5S*, trnA*, *trnA-UGC*, trnE-UUC*, *trnH-CAU*, *trnI-CAU*, trnI-GAU*, *trnL-CAA*(×2), *trnM*, *trnM-CAU*, *trnN*, *trnN-GUU*, *trnR*, *trnR-ACG*(×2), *ycf15*(×2), *ycf2*, *ycf68*
IRB	*rpI23*, *rrn16*, *rrn23*, *rrn23S*, *rrn5*, *rrn5S*, trnE-UUC*, *trnH-AUG*, *trnH-CAU*, *trnI-CAU*, trnI-GAU*, *trnM*, *trnM-CAU*, *trnN*, *trnN-GUU*, *trnR*, *trnR-ACG*(×2), *trnV*, *trV-GAC*, *ycf15*, *ycf2*, *ycf68*
LSC	*accD*, *atpA*, *atpB*, *atpE*, atpF*, *atpH*, *atpI*, *cemA*, clpP1*, *IhbA*, *infA*, *matK*, *ndhC*, *ndhJ*, *ndhK*, *pbf1*, *petA*, *petB*, *petD*, *petG*, *petL*, *petN*, *psaA*, *psaB*, *psaI*(×2), *psbA*, *psbB*, *psbC*, *psbD*, *psbE*, *psbF*, *psbH*, *psbI*, *psbJ*, *psbK*, *psbL*, *psbM*, *psbT*, *psbZ*, *rbcL*, *rpI14*, *rpI16*, rpI2*, *rpI22*, *rpI33*, *rpI36*, *rpl20*, *rpoA*, *rpoB*, rpoC1*, *rpoC2*, *rps11*, *rps12*, *rps14*, rps16*, *rps18*, *rps19*, *rps2*, *rps3*, *rps4*, *rps8*, *trnA-FME*, *trnC*, *trnC-GCA*, *trnD-GUC*(×2), trnE-UUC*, *trnF*, *trnF-GAA*(×2), *trnfM*, *trnG*, *trnG-GCC*(×2), trnG-GCC*, *trnG-UCC*, trnG-UCC*, *trnH*, *trnH-GUG*(×2), trnK-UUU*, trnL-UAA*, *trnM*(×2), *trnM-CAU*(×2), *trnP*, *trnP-GGG*, *trnP-UGG*, *trnQ*, *trnQ-UUG*, *trnR*, *trnR-UCU*, *trnS*(×2), *trnS-GCU*, *trnS-GGA*, *trnS-UGA*(×2), trnT-CGU*, *trnT-GGU*(×2), *trnT-UGU*, *trn-UGG*, trnV-UAC*, *trnW*, *trnW-CCA*, *trnY-GUA*, *ycf3*, *ycf4*

*: Gene containing a single intron; (×2): two gene copies in the IRs.

**Table 3:** List of genes encoded in the chloroplast genome of HCNC.

Category for Genes	Group of Genes	Name of Genes
Photosynthesis-related genes	Rubisco	*rbcL*
	Photosystem I	*psaA*, *psaB*, *psaC*, *psaI*, *psaJ*
	Assembly/stability of photosystem I	ycf3*, *ycf4*
	Photosystem II	*psbA*, *psbB*, *psbC*, *psbD*, *psbE*, *psbF*, *psbH*, *psbI*, *psbJ*, *psbK*, *psbL*, *psbM*, *psbT*, *psbZ*
	ATP synthase	*atpA*, *atpB*, *atpE*, *atpF*, *atpH*, *atpI*
	Cytochrome b/f complex	*petA*, *petB*, *petD*, *petG*, *petL*, *petN*
	Cytochrome c synthesis	*ccsA*
	NADPH dehydrogenase	*ndhA*, *ndhB*(×2), *ndhC*, *dnhD*, *ndhF*, *ndhG*, *ndhH*, *ndhI*, *ndhJ*, *ndhK*
Transcription and translation-related genes	Transcription	*rpoA*, *rpoB*, *rpoC1*, *rpoC2*
	Ribosomal proteins	*rps2*, *rps3*, *rps4*, *rps7*(×2), *rps8*, *rps11*, *rps12*, *rps14*, *rps15*, *rps16*, *rps18*, *rps19*
	Translation initiation factor	*infA*
RNA genes	Ribosomal RNA	*rrn4.5*, *rrn5*(×2), *rrn16*(×2), *rrnS23*(×2)
	Transfer RNA	trnA-UGC*(×2), *trnC-GCA*, *trnD-GUC*(×2), trnE-UUC*(×2), *trnF-GAA*(×2), *trnG-GCC*(×2), *trnG-UCC*(×2), *trnH-AUG*, *trnH-CAU*(×2), *trnH-GUG*(×2), *trnI-CAU*(×2), trnI-GAU*(×2), *trnK-UUU*(×2), *trnL-CAA*(×2), trnL-UAA*, *trnM-CAU*(×2), *trnN-GUU*(×2), *trnP-GGG*, *trnP-UGG*(×2), *trnQ-UUG*, *trnR-ACG*(×2), *trnR-UCU*, *trnS-GCU*, *trnS-GGA*, *trnS-UGA*(×2), *trnT-CGU*, *trnT-GGU*, *trnT-UGU*, *trnV-GAC*(×2), trnV-UAC*, *trnW-CCA*, *trnY-GUA*
Other genes	RNA processing	*matK*
	Carbon metabolism	*cemA*
	Fatty acid synthesis	*accD*
	Proteolysis	*clpP1*
Genes of unknown function	Conserved reading frames	*lhbA*, *pbf1*, *ycf1*, *ycf2*(×2), *ycf 8*, *ycf15*(×2)

*: Gene containing a single intron; (×2): two gene copies in the IRs.

**Table 4:** List of InDels in cp genomes of seven *C. sinensis* var. *sinensis*.

No. of InDels	HCNC	Beachwisull	Keumsull	Chinese Wild Type	Saemidori	Fushun	Hadong Wild Type
Homo	2,470,948	2,424,703	2,392,460	1,798,551	2,524,129	2,380,977	2,398,986
Hetero	2,602,999	2,765,874	2,864,054	4,098,625	2,503,503	2,920,008	2,823,932
Total	5,073,947	5,190,577	5,256,514	5,897,176	5,027,632	5,300,985	5,222,918

**Table 5:** List of SNPs in cp genomes of seven *C. sinensis* var. *sinensis*.

No. of SNPs	HCNC	Beachwisull	Keumsull	Chinese Wild Type	Saemidori	Fushun	Hadong Wild Type
Homo	24,012,532	23,473,163	23,096,147	16,262,147	24,653,657	23,069,934	23,124,571
Hetero	50,530,416	52,831,530	54,347,952	73,348,257	49,275,996	56,236,149	53,599,523
Total	74,542,948	76,304,693	77,444,099	89,610,582	73,929,653	79,306,083	76,824,094

**Table 6:** The distribution of the identified SNP in the different genomic regions (intergenic, upstream, downstream, intron, exon, and others) of the HCNC.

Type (Alphabetical Order)	Count	Percentage (%)
Downstream	21,271,665	8.77
Exon	3,465,984	1.43
Intergenic	174,996,970	72.13
Intron	16,660,026	6.87
None	2,886,144	1.19
Splice site acceptor	18,235	0.01
Splice site donor	13,084	0.01
Splice site region	153,994	0.06
Transcript	3991	0.00
Upstream	22,283,348	9.19
UTR 3 prime	431,832	0.18
UTR 5 prime	416,661	0.17

## Data Availability

Data are contained within the article.

## References

[1] Zhang X., Chen S., Shi L., Gong D., Zhang S., Zhao Q., Zhan D., Vasseur L., Wang Y., Yu J. (2021). Haplotype-resolved genome assembly provides insights into evolutionary history of the tea plant *Camellia sinensis*. Nat. Genet..

[2] Li L., Hu Y., He M., Zhang B., Wu W., Cai P., Huo D., Hong Y. (2021). Comparative chloroplast genomes: Insights into the evolution of the chloroplast genome of *Camellia sinensis* and the phylogeny of Camellia. BMC Genom..

[3] Kumarihami H.P.C., Oh E.U., Nesumi A., Song K.J. (2016). Comparative study on cross-compatibility between *Camellia sinensis* var. *sinensis* (China type) and *C. sinensis* var. *assamica* (Assam type) tea. Afr. J. Agric. Res..

[4] Meegahakumbura M.K., Wambulwa M.C., Li M.M., Thapa K.K., Sun Y.S., Moller M., Xu J.C., Yang J.B., Liu J., Liu B.Y. (2017). Domestication Origin and Breeding History of the Tea Plant (*Camellia sinensis*) in China and India Based on Nuclear Microsatellites and cpDNA Sequence Data. Front. Plant Sci..

[5] Cho K.H., Lee E.J., Tsuge T., Jo A., Kim J.C., Cheong G.W., Yoon H.S., Kim G.T. (2010). Comparative genomic analysis of Korean and Japanese green tea. Can. J. Plant Sci..

[6] Choi K.S., Chung M.G., Park S. (2016). The Complete Chloroplast Genome Sequences of Three Veroniceae Species (Plantaginaceae): Comparative Analysis and Highly Divergent Regions. Front. Plant Sci..

[7] Xia E.H., Tong W., Wu Q., Wei S., Zhao J., Zhang Z.Z., Wei C.L., Wan X.C. (2020). Tea plant genomics: Achievements, challenges and perspectives. Hortic. Res..

[8] Daniell H., Jin S., Zhu X.G., Gitzendanner M.A., Soltis D.E., Soltis P.S. (2021). Green giant-a tiny chloroplast genome with mighty power to produce high-value proteins: History and phylogeny. Plant Biotechnol. J..

[9] Zhu B., Qian F., Hou Y., Yang W., Cai M., Wu X. (2021). Complete chloroplast genome features and phylogenetic analysis of Eruca sativa (Brassicaceae). PLoS ONE.

[10] Cui Y., Chen X., Nie L., Sun W., Hu H., Lin Y., Li H., Zheng X., Song J., Yao H. (2019). Comparison and Phylogenetic Analysis of Chloroplast Genomes of Three Medicinal and Edible Amomum Species. Int. J. Mol. Sci..

[11] Li H., Song K., Zhang X., Wang D., Dong S., Liu Y., Yang L. (2023). Application of Multi-Perspectives in Tea Breeding and the Main Directions. Int. J. Mol. Sci..

[12] Jansen R.K., Raubeson L.A., Boore J.L., dePamphilis C.W., Chumley T.W., Haberle R.C., Wyman S.K., Alverson A.J., Peery R., Herman S.J. (2005). Methods for obtaining and analyzing whole chloroplast genome sequences. Methods Enzym..

[13] Olmstead R.G., Palmer J.D. (1994). Chloroplast dna systematics: A review of methods and data analysis. Am. J. Bot..

[14] Zhang W., Zhao Y., Yang G., Peng J., Chen S., Xu Z. (2019). Determination of the evolutionary pressure on Camellia oleifera on Hainan Island using the complete chloroplast genome sequence. PeerJ.

[15] Shen X., Guo S., Yin Y., Zhang J., Yin X., Liang C., Wang Z., Huang B., Liu Y., Xiao S. (2018). Complete Chloroplast Genome Sequence and Phylogenetic Analysis of Aster tataricus. Molecules.

[16] Wicke S., Schneeweiss G.M., dePamphilis C.W., Muller K.F., Quandt D. (2011). The evolution of the plastid chromosome in land plants: Gene content, gene order, gene function. Plant Mol. Biol..

[17] Corriveau J.L., Coleman A.W. (1988). Rapid screening method to detect potential biparental inheritance of plastid dna and results for over 200 angiosperm species. Am. J. Bot..

[18] Sugita M., Sugiura M. (1996). Regulation of gene expression in chloroplasts of higher plants. Plant Mol. Biol..

[19] Zoclanclounon Y.A.B., Thamilarasan S.K., Mo Y., Ahn B.O., Kim J.G., Lee K. (2023). Insights into chloroplast genome structure and phylogenetic relationships within the Sesamum species complex (Pedaliaceae). Front. Genet..

[20] Liu S., Liu H., Wu A., Hou Y., An Y., Wei C. (2017). Construction of fingerprinting for tea plant (*Camellia sinensis*) accessions using new genomic SSR markers. Mol. Breed..

[21] Wang C., Han J., Pu Y., Wang X. (2022). Tea (*Camellia sinensis*): A Review of Nutritional Composition, Potential Applications, and Omics Research. Appl. Sci..

[22] Lee D.J., Kim C.K., Lee T.H., Lee S.J., Moon D.G., Kwon Y.H., Cho J.Y. (2020). The complete chloroplast genome sequence of economical standard tea plant, *Camellia sinensis* L. cultivar Sangmok, in Korea. Mitochondrial DNA B Resour..

[23] Zhang W., Zhang Y., Qiu H., Guo Y., Wan H., Zhang X., Scossa F., Alseekh S., Zhang Q., Wang P. (2020). Genome assembly of wild tea tree DASZ reveals pedigree and selection history of tea varieties. Nat. Commun..

[24] Greiner S., Lehwark P., Bock R. (2019). OrganellarGenomeDRAW (OGDRAW) version 1.3.1: Expanded toolkit for the graphical visualization of organellar genomes. Nucleic Acids Res..

[25] Ma P.F., Zhang Y.X., Zeng C.X., Guo Z.H., Li D.Z. (2014). Chloroplast phylogenomic analyses resolve deep-level relationships of an intractable bamboo tribe Arundinarieae (poaceae). Syst. Biol..

[26] Rawal H.C., Borchetia S., Bera B., Soundararajan S., Ilango R.V.J., Barooah A.K., Sharma T.R., Singh N.K., Mondal T.K. (2021). Comparative analysis of chloroplast genomes indicated different origin for Indian tea (*Camellia assamica* cv TV1) as compared to Chinese tea. Sci. Rep..

[27] Zhang Y., Du L., Liu A., Chen J., Wu L., Hu W., Zhang W., Kim K., Lee S.C., Yang T.J. (2016). The Complete Chloroplast Genome Sequences of Five Epimedium Species: Lights into Phylogenetic and Taxonomic Analyses. Front. Plant Sci..

[28] Zhang Y.J., Ma P.F., Li D.Z. (2011). High-throughput sequencing of six bamboo chloroplast genomes: Phylogenetic implications for temperate woody bamboos (Poaceae: Bambusoideae). PLoS ONE.

[29] Yang J.B., Tang M., Li H.T., Zhang Z.R., Li D.Z. (2013). Complete chloroplast genome of the genus Cymbidium: Lights into the species identification, phylogenetic implications and population genetic analyses. BMC Evol. Biol..

[30] Yang M., Zhang X., Liu G., Yin Y., Chen K., Yun Q., Zhao D., Al-Mssallem I.S., Yu J. (2010). The complete chloroplast genome sequence of date palm (*Phoenix dactylifera* L.). PLoS ONE.

[31] Huang C., Zhang J., Zhang X., Yu Y., Bian W., Zeng Z., Sun X., Li X. (2018). Two New Polyphenol Oxidase Genes of Tea Plant (*Camellia sinensis*) Respond Differentially to the Regurgitant of Tea Geometrid, Ectropis obliqua. Int. J. Mol. Sci..

[32] Park I., Yang S., Kim W.J., Song J.H., Lee H.S., Lee H.O., Lee J.H., Ahn S.N., Moon B.C. (2019). Sequencing and Comparative Analysis of the Chloroplast Genome of Angelica polymorpha and the Development of a Novel Indel Marker for Species Identification. Molecules.

[33] Zan T., He Y.T., Zhang M., Yonezawa T., Ma H., Zhao Q.M., Kuo W.Y., Zhang W.J., Huang C.H. (2023). Phylogenomic analyses of Camellia support reticulate evolution among major clades. Mol. Phylogenet Evol..

[34] Song W., Chen Z., Shi W., Han W., Feng Q., Shi C., Engel M.S., Wang S. (2022). Comparative Analysis of Complete Chloroplast Genomes of Nine Species of Litsea (Lauraceae): Hypervariable Regions, Positive Selection, and Phylogenetic Relationships. Genes.

[35] Zhai W., Duan X., Zhang R., Guo C., Li L., Xu G., Shan H., Kong H., Ren Y. (2019). Chloroplast genomic data provide new and robust insights into the phylogeny and evolution of the Ranunculaceae. Mol. Phylogenet Evol..

[36] Petersen G., Aagesen L., Seberg O., Larsen I.H. (2011). When is enough, enough in phylogenetics? A case in point from Hordeum (Poaceae). Cladistics.

[37] Lu Q.X., Chang X., Gao J., Wu X., Wu J., Qi Z.C., Wang R.H., Yan X.L., Li P. (2022). Evolutionary Comparison of the Complete Chloroplast Genomes in Convallaria Species and Phylogenetic Study of Asparagaceae. Genes.

[38] Hazra A., Mahadani P., Das S., Bhattacharya S., Kumar R., Sengupta C., Das S. (2020). Insight to the ancestral relations and varietal diversity of Indian tea [*Camellia sinensis* (L.) Kuntze] through plastid and nuclear phylogenetic markers. Genet. Resour. Crop Evol..

[39] Chen X., Li B., Zhang X. (2023). Comparison of chloroplast genomes and phylogenetic analysis of four species in Quercus section Cyclobalanopsis. Sci. Rep..

[40] Wu Q., Tong W., Zhao H., Ge R., Li R., Huang J., Li F., Wang Y., Mallano A.I., Deng W. (2022). Comparative transcriptomic analysis unveils the deep phylogeny and secondary metabolite evolution of 116 Camellia plants. Plant J..

[41] Ryu Y., Kim I.R., Su M.H., Jung J., Choi H.-K., Kim C. (2019). Phylogeographical Study of Camellia japonica Inferred from AFLP and Chloroplast DNA Haplotype Analyses. J. Plant Biol..

